# Supplementary material for: Efficacy of Repetitive Transcranial Magnetic Stimulation (rTMS) Combined with Psychological Interventions: A Systematic Review and Meta-Analysis of Randomized Controlled Trials
Source: Brain Sci. 2023 Nov 30;13(12):1665. doi: 10.3390/brainsci13121665 (PMC10741493; doi:10.3390/brainsci13121665)
Supplement: Supplementary file 1 [file brainsci-13-01665-s001.zip › brainsci-2712370-supplementary/brainsci-2712370-supplementary.pdf]

Supplementary files

Table S1. Search Strategies.

Database	Search strategy
PubMed	("Transcranial Magnetic Stimulation"[Mesh] OR "transcranial magnetic stimulations" OR "TMS" OR "transcranial magnetic stimulation" OR "rTMS" OR "theta burst stimulation" OR "TBS") AND ("Psychotherapy"[Mesh] OR psychotherapy OR psychotherapies OR "psychotherapeutic counseling" OR "psychotherapeutic counselling" OR "psychotherapeutic processes" OR "psychotherapeutic training" OR "psychotherapeutic treatment" OR "psychotherapeutic treatments" OR psychologic OR psychological OR psychologically OR "psychosocial treatment" OR "psychosocial treatments" OR "Behavior Therapy"[Mesh] OR "behavior modification" OR "behavior regulation" OR "behavior therapy" OR "behavior therapies" OR "behaviour modification" OR "behaviour regulation" OR "behaviour therapy" OR "behaviour therapies" OR "Cognitive Behavioral Therapy"[Mesh] OR CBT OR "cognitive behavior" OR "cognitive behaviorial" OR "Cognitive Remediation"[Mesh] OR "cognitive intervention" OR "cognitive interventions" OR "cognitive rehabilitation" OR "cognitive training" OR "cognitive trainings" OR "cognitive remediation" OR "cognitive technique" OR "cognitive techniques" OR "cognitive therapy" OR "cognitive therapies" OR "cognitive treatment" OR "cognitive treatments" OR "Counseling"[Mesh] OR counseling OR counselling OR "Association"[Mesh] OR "Association Learning"[Mesh] OR “Mindfulness”[Mesh] OR “Meditation”[Mesh] OR meditation OR mindfulness OR "exposure therapy" OR "exposure therapies" OR "family intervention" OR "family interventions" OR "family therapy" OR "family therapies" OR "group intervention" OR "group interventions" OR "group psychotherapy" OR "group psychotherapies" OR "group therapy" OR "group therapies" OR psychoeducate OR psychoeducation OR psychoeducating) AND ((randomized controlled trial[Publication Type]) OR (controlled clinical trial[Publication Type]) OR (randomized[Title/Abstract]) OR (randomised[Title/Abstract]) OR (randomly[Title/Abstract]) OR (trial[Title/Abstract])) NOT ((animals [mh] NOT humans [mh])) AND (English[Language])
Ovid EMBASE	1 exp Transcranial Magnetic Stimulation/<br>2 (transcranial magnetic stimulations or TMS or transcranial magnetic stimulation or rTMS or theta burst stimulation or TBS).ti,ab,kf.<br>3 1 or 2<br>4 exp Psychotherapy/<br>5 exp Behavior Therapy/<br>6 exp Cognitive Behavioral Therapy/<br>7 exp Cognitive Remediation/<br>8 exp Counseling/<br>9 exp Association Learning/ or exp Association/<br>10 exp Mindfulness/<br>11 exp Meditation /

	12 4 or 5 or 6 or 7 or 8 or 9 or 10 or 11<br>13 (psychotherapy or psychotherapies or psychotherapeutic counseling or psychotherapeutic counselling or psychotherapeutic processes or psychotherapeutic training or psychotherapeutic treatment or psychotherapeutic treatments or psychologic or psychological or psychologically or psychosocial treatment or psychosocial treatments or behavior modification or behavior regulation or behavior therapy or behavior therapies or behaviour modification or behaviour regulation or behaviour therapy or behaviour therapies or CBT or cognitive behavior or cognitive behaviorial or cognitive intervention or cognitive training or cognitive trainings or cognitive interventions or cognitive rehabilitation or cognitive remediation or cognitive technique or cognitive techniques or cognitive therapy or cognitive therapies or cognitive treatment or cognitive treatments or counseling or counselling or meditation or mindfulness or exposure therapy or exposure therapies or family intervention or family interventions or family therapy or family therapies or group intervention or group interventions or group psychotherapy or group psychotherapies or group therapy or group therapies or psychoeducate or psychoeducation or psychoeducating). ti,ab,kf.<br>14 12 or 13<br>15 ('crossover procedure':de or 'double-blind procedure':de or 'randomized controlled trial':de or 'single-blind procedure':de).mp. or (random* or factorial* or crossover* or cross NEXT over* or placebo* or doubl* NEAR blind* or singl* NEAR blind* or assign* or allocat* or volunteer*).de,ab,ti.<br>16 3 and 14 and 15<br>limit 16 to english language and human
PsycINFO	1 su(Transcranial Magnetic Stimulation)<br>2 TIABSU("transcranial magnetic stimulations" or "TMS" or "transcranial magnetic stimulation" or "rTMS" or "theta burst stimulation" or "TBS")<br>3 1 OR 2<br>4 su(Psychotherapy) OR su(Behavior Therapy) OR su(Cognitive Behavioral Therapy) OR su(Cognitive Remediation) OR su(Counseling) OR su(Association Learning) OR su(Association) OR su(Mindfulness) OR su(Meditation)<br>5 tiabsu(psychotherapy or psychotherapies or psychotherapeutic counseling or psychotherapeutic counselling or psychotherapeutic processes or psychotherapeutic training or psychotherapeutic treatment or psychotherapeutic treatments or psychologic or psychological or psychologically or psychosocial treatment or psychosocial treatments or behavior modification or behavior regulation or behavior therapy or behavior

		therapies or behaviour modification or behaviour regulation or behaviour therapy or behaviour therapies or CBT or cognitive behavior or cognitive behaviorial or cognitive intervention or cognitive training or cognitive trainings or cognitive interventions or cognitive rehabilitation or cognitive remediation or cognitive technique or cognitive techniques or cognitive therapy or cognitive therapies or cognitive treatment or cognitive treatments or counseling or counselling or meditation or mindfulness or exposure therapy or exposure therapies or family intervention or family interventions or family therapy or family therapies or group intervention or group interventions or group psychotherapy or group psychotherapies or group therapy or group therapies or psychoeducate or psychoeducation or psychoeducating)
	6	4 OR 5
	7	SU.EXACT("Treatment Effectiveness Evaluation") OR SU.EXACT.EXPLODE("Treatment Outcomes") OR SU.EXACT("Placebo") OR SU.EXACT("Followup Studies") OR placebo* OR random* OR "comparative stud*" OR clinical NEAR/3 trial* OR research NEAR/3 design OR evaluat* NEAR/3 stud* OR prospectiv* NEAR/3 stud* OR (singl* OR doubl* OR trebl* OR tripl*) NEAR/3 (blind* OR mask*)
	8	3 AND 6 AND 7
	9	Limited 8 to English
Cochrane Library	#1	MeSH descriptor: [Transcranial Magnetic Stimulation] explode all trees
	#2	(“transcranial magnetic stimulations”):ti,ab,kw OR (“TMS”):ti,ab,kw OR (“transcranial magnetic stimulation”):ti,ab,kw OR (“rTMS”):ti,ab,kw (Word variations have been searched)
	#3	(“theta burst stimulation”):ti,ab,kw OR (“TBS”):ti,ab,kw (Word variations have been searched)
	#4	#1 OR #2 OR #3
	#5	MeSH descriptor: [Psychotherapy] explode all trees
	#6	MeSH descriptor: [Behavior Therapy] explode all trees
	#7	MeSH descriptor: [Cognitive Behavioral Therapy] explode all trees
	#8	MeSH descriptor: [Cognitive Remediation] explode all trees
	#9	MeSH descriptor: [Counseling] explode all trees
	#10	MeSH descriptor: [Association Learning] explode all trees
	#11	MeSH descriptor: [Association] explode all trees
	#12	MeSH descriptor: [Mindfulness] explode all trees
	#13	MeSH descriptor: [Meditation] explode all trees
	#14	#5 OR #6 OR #7 OR #8 OR #9 OR #10 OR #11 OR #12 OR #13
	#15	("psychotherapy" or "psychotherapies" or "psychotherapeutic counseling" or "psychotherapeutic counselling" or "psychotherapeutic processes" or "psychotherapeutic training" or "psychotherapeutic treatment" or "psychotherapeutic treatments" or "psychologic" or "psychological" or "psychologically" or "psychosocial treatment" or "psychosocial treatments" or "behavior

	modification" or "behavior regulation" or "behavior therapy" or "behavior therapies" or "behaviour modification" or "behaviour regulation" or "behaviour therapy" or "behaviour therapies" or "CBT" or "cognitive behavior" or "cognitive behaviorial" or "cognitive intervention" or "cognitive training" or "cognitive trainings" or "cognitive interventions" or "cognitive rehabilitation" or "cognitive remediation" or "cognitive technique" or "cognitive techniques" or "cognitive therapy" or "cognitive therapies" or "cognitive treatment" or "cognitive treatments" or "counseling" or "counselling" or "meditation" or "mindfulness" or "exposure therapy" or "exposure therapies" or "family intervention" or "family interventions" or "family therapy" or "family therapies" or "group intervention" or "group interventions" or "group psychotherapy" or "group psychotherapies" or "group therapy" or "group therapies" or "psychoeducate" or "psychoeducation" or "psychoeducating"):ti,ab,kw (Word variations have been searched)<br>#16 #14 OR #15<br>#17 (randomized controlled trial):pt OR (controlled clinical trial):pt OR (randomized):ti,ab,kw (Word variations have been searched)<br>#18 (randomised):ti,ab,kw OR (randomly):ti,ab,kw OR (trial):ti,ab,kw (Word variations have been searched)<br>#19 #17 OR #18<br>#20 #4 AND #16 AND #19 in Trials

Table S2. Clinical symptoms variables included in the analyses.

Variables	Questionnaires	k
Clinical symptoms	CAPS	4
	AQ	1
	PAS	1
	PANAS	1
	QSU	1
	CAARS	1
	FTND	2
	NPI	1
	OCDS	1
	Y-BOCS	1
	HAMD-17	1
	HDRS-17	1
Depressive symptoms	BDI	1
	GDS	2
	PHQ-9	1
	HAMD-17	1
	HDRS	1
	HDRS-17	1
	HDRS-21	1
	HDRS-24	1
	QIDS	2

Note: AQ: Acrophobia Questionnaire; BDI: Beck's Depression Inventory; CAARS: Conners' Adult ADHD Rating Scale; CAPS: Clinician-Administered PTSD Scale; FTND: Fagerström Test for Nicotine Dependence; GDS: Yesavage's Geriatric Depression Scale; HAMD: Hamilton Depression Rating Scale; HDRS: Hamilton Depression Rating Scale; NPI: Neuropsychiatric Inventory; OCDS: Obsessive compulsive drinking scale; PANAS: Positive and Negative Affect Scale; PAS: Panic and Agoraphobia Scale; PHQ-9: patient health questionnaire-9; QIDS: Quick Inventory of Depressive Symptomatology; QSU: Questionnaire on Smoking Urges; Y-BOCS: Yale-Brown-Obsessive-Compulsive Scale.

Table S3. Functional outcomes variables included in the analyses.

Variables	Questionnaires	k
Social and vocational functioning	IPF	1
	WSA	1
Daily activities	IWI	1
	FIM	1
	ADL	2
	MBI	1
Quality of life	AAQoL	1

Note: AAQOL: Adult ADHD Quality of Life; ADL: activities of daily living; FIM: Functional Independence Measure; IPF: Inventory of Psychosocial Functioning; IWI: interview with informant; MBI: Modified Barthel Index; WSAS: Work and Social Adjustment Scale.

Table S4. Cognitive variables included in the analyses.

Cognitive domain	Cognitive tasks or questionnaires	k
Global cognition	ADAS-Cog	3
	LOTCA	1
	Mindstreams global score	1
	MMSE	5
	MoCA	2
	Raven's progressive matrices (accuracy)	2
Working memory	3-Back task (accuracy)	1
	Digit span task (backward span)	1
	Maastricht working memory training program	1
	NIH Examiner n-back (2 back)	1
	NIH Examiner Dot Counting	1
Executive function	BRIEF-A Global Executive Composite	1
	Stroop (effect time)	1
	Stop-switching task (SSRT)	1
	Go/no-go (RT)	1
Complex attention	Digit detection	1
	Reaction time tasks (simple, direction choice and color choice) (RT)	1
	Attention matrices	1
	DST	1
	Visual attention (RT)	1
	Digit span task (forward span)	1
	TMT-A (RT)	2
	ACE-III (attention)	1
	DS (forward and backward)	1
Language	Language (phonemic and semantic verbal fluency)	1
	ACE-III (verbal fluency and language)	1
Learning and memory	ROCF	1
	ROCF, delayed recall	1
	Face-name associative memory	1
	RAVLT (delayed and immediate recall)	1
	Story recall	1
	ACE-III (memory)	1
Perceptual-motor function	Mental rotation (score)	1
	ROCF, copy	1

Note: ACE-III: Addenbrooke's Cognitive Examination-III; ADAS-Cog: Alzheimer's Disease Assessment scale in cognitive subdomain; BRIEF-A: Behavior Rating Inventory of Executive Function-Adult Version; ; DS: Digital Span Test; DST: Digit Symbol Test; LOTCA: loewenstein occupational therapy cognitive assessment; MMSE: Mini-Mental State Examination; MoCA: Montreal Cognitive Assessment; NIH: National Institute of Health; SSRT: stop signal reaction time; RT: reaction time; RAVLT, Rey Auditory Verbal Learning Test; ROCF: Rey-Osterrieth Complex Fig. test; TMT-A: Trail Making Test-A.

Table S5. Risk of bias assessment.

Study	Signalling questions	Response	Description
Herrmann et al. (2017)	1.1 Was the allocation sequence random?	NI	Quote: "Participants meeting the inclusion criteria were randomly (simple randomization 1-1; controlling for the factor sex) assigned to two groups (active vs. sham)."
	1.2 Was the allocation sequence concealed until participants were enrolled and assigned to interventions?	NI	No information about concealing the allocation was given in the paper.
	1.3 Did baseline differences between intervention groups suggest a problem with the randomization process?	PY	The baseline scores of the Attitude Towards Heights Questionnaire and Anxiety-Sensitivity Index 3 were significantly different between patients with height phobia in active and sham groups.
Osuch et al. (2009)	2.1. Were participants aware of their assigned intervention during each period of the trial?	N	Quote: "Neither the patient nor the researcher assessing symptoms knew which phase was active."
	2.2. Were carers and people delivering the interventions aware of participants' assigned intervention during each period of the trial?	Y	Quote: "The individual administering the rTMS was not blind to phase."
	2.3. [if applicable] If Y/PY/NI to 2.1 or 2.2: Were important non-protocol-interventions balanced between interventions?	NI	No counterbalance information was provided.
	2.4. [if applicable] Were there failures in implementing the intervention that could have affected the outcome?	NA	
	2.5. [if applicable] Was there non-adherence to the assigned intervention regimen that could have affected participants' outcomes?	NA	

	2.6. If N/PN/NI to 2.3, 2.4, or 2.5: Was an appropriate analysis used to estimate the effect of adhering to the intervention?	PN	Quote: “The individual administering the rTMS was not blind to phase.”; “Given the small sample size of this preliminary study, other statistics were not performed”. The author probably didn’t use appropriate analysis strategies to estimate the effect of adhering to intervention.

Note: NI: No information; PY: Probably yes; N: No; Y: Yes; NA: Not applicable; PN: Probably no.

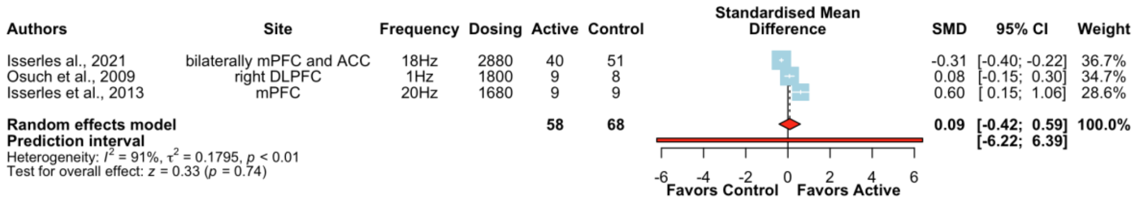

Figure S1. Forest plots of the effect of rTMS + exposure therapy on clinical symptoms in patients with post-traumatic stress disorder (PTSD). Note: ACC: anterior cingulate cortex; CI: confidence interval; DLPFC: dorsolateral prefrontal cortex; mPFC: medial prefrontal cortex; SMD: standardized mean differences.

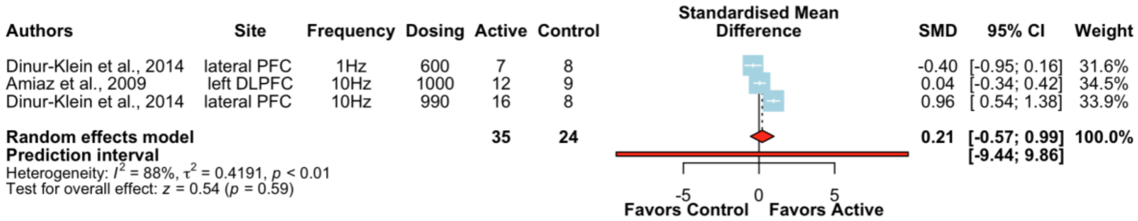

Figure S2. Forest plots of the effect of rTMS + exposure therapy on clinical symptoms in smokers. Note: PFC: prefrontal cortex.

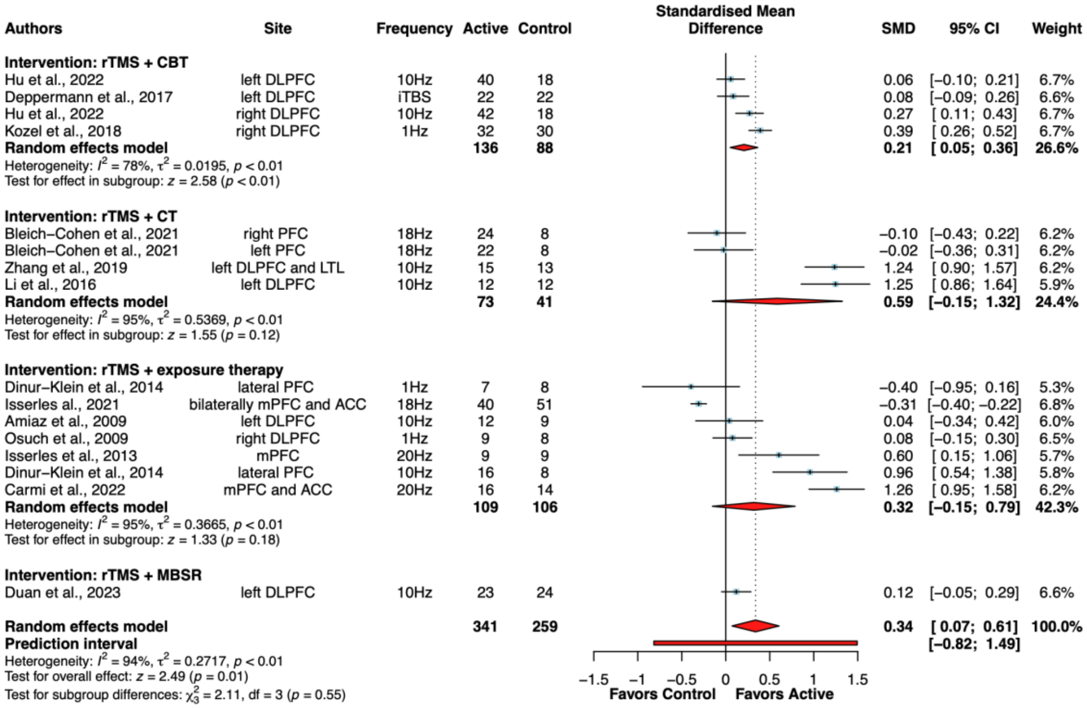

Figure S3. Forest plots of the effect of rTMS + psychological interventions with 10 or more sessions on clinical symptoms. Note: LTL: lateral temporal lobe.

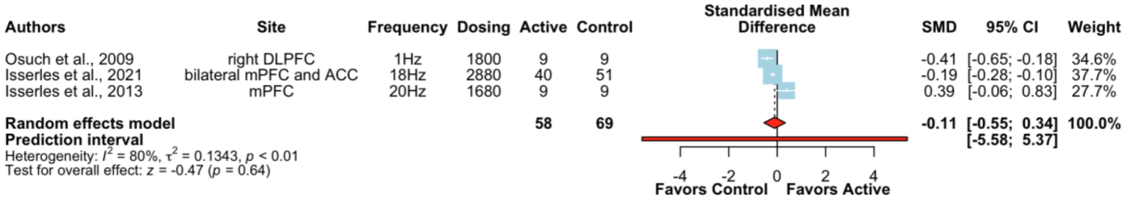

Figure S4. Forest plots of the effect of rTMS + exposure therapy on depressive symptoms in patients with PTSD.

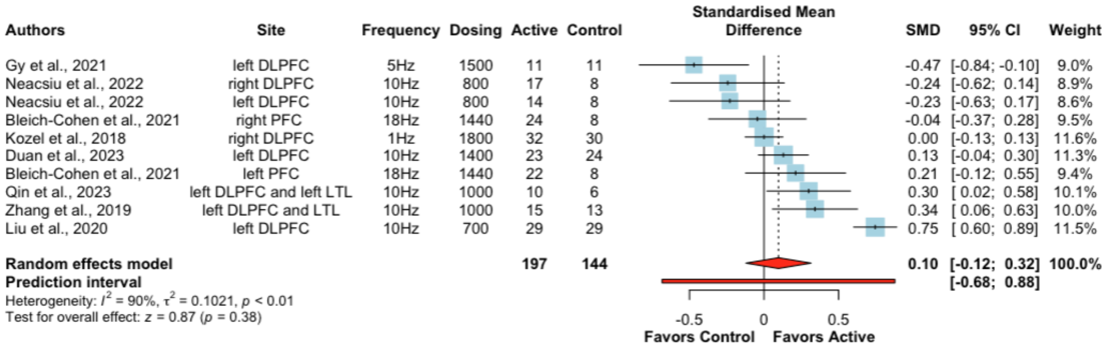

Figure S5. Forest plots of the effect of rTMS + psychological interventions on functional outcomes.

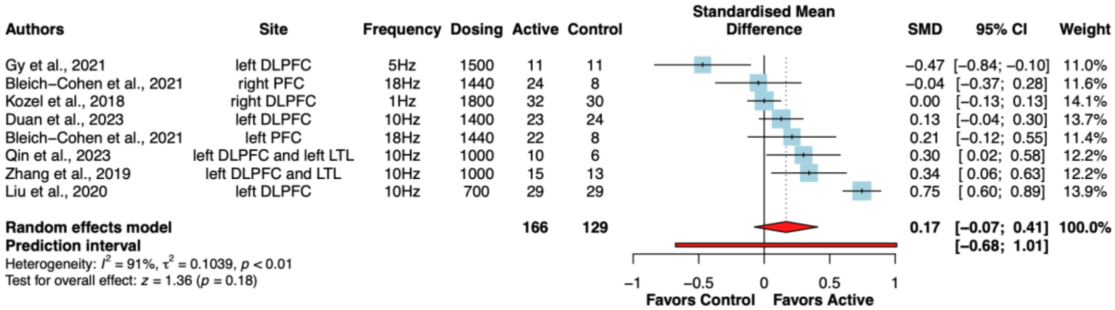

Figure S6. Forest plots of the effect of rTMS + psychological interventions with 10 or more sessions on functional outcomes.

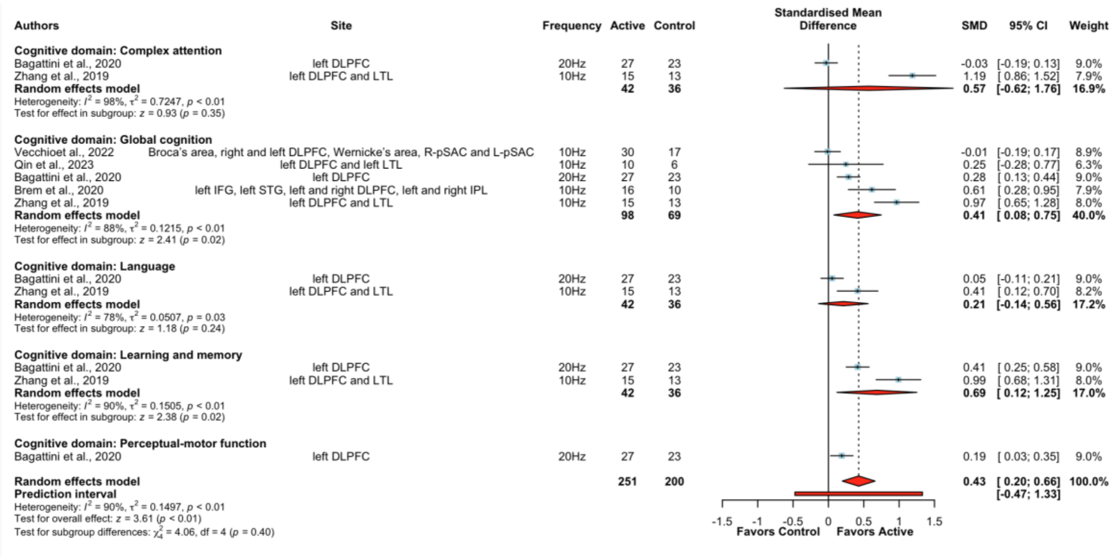

Figure S7. Forest plots of the effect of rTMS + CT for different cognitive domains in patients with AD. Note: IFG: inferior frontal gyrus; IPL: inferior parietal lobule; pSAC: parietal somatosensory association cortices; STG: superior temporal gyrus.

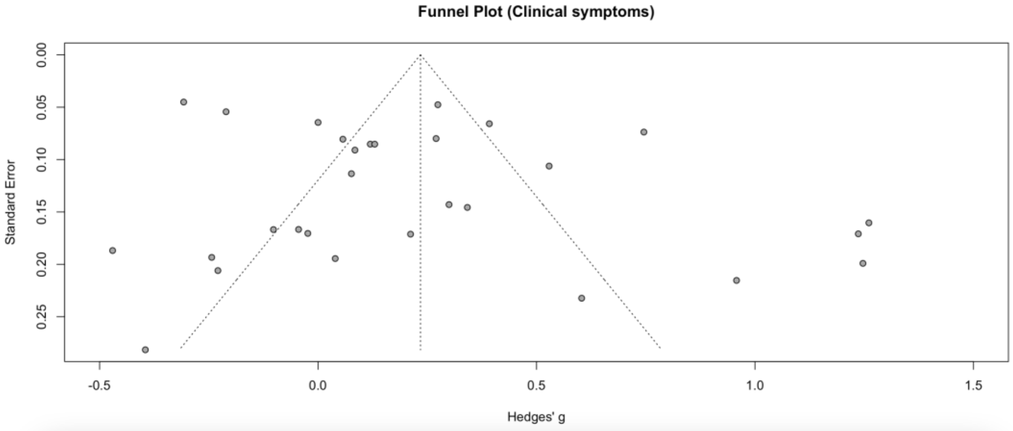

Figure S8. Funnel plot for the effect of clinical symptoms.

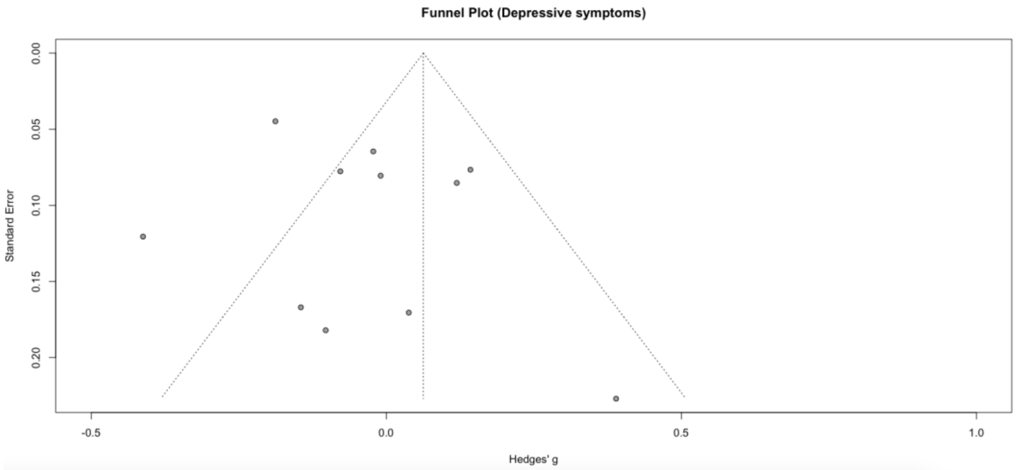

Figure S9. Funnel plot for the effect of depressive symptoms.

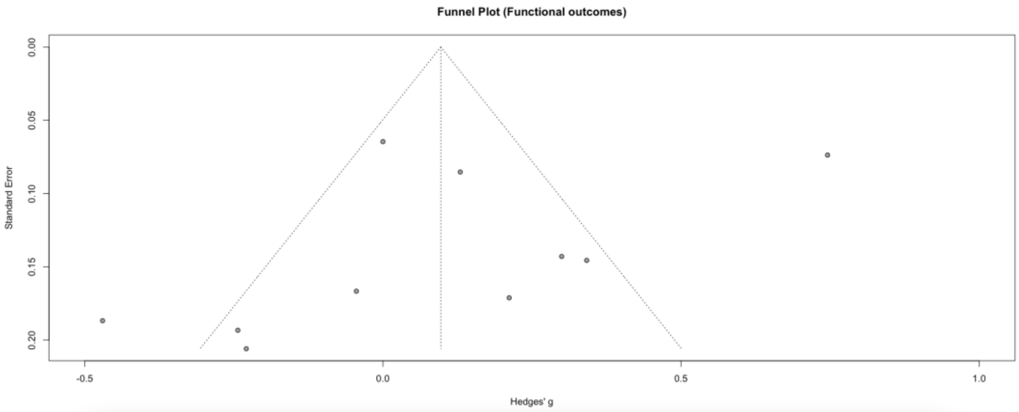

Figure S10. Funnel plot for the effect of functional outcomes.

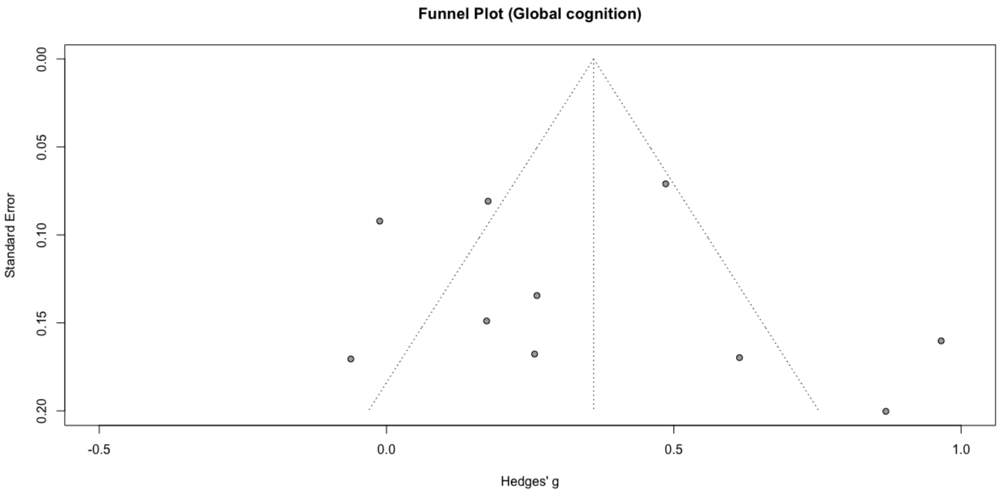

Figure. S11. Funnel plot for the effect of global cognition.

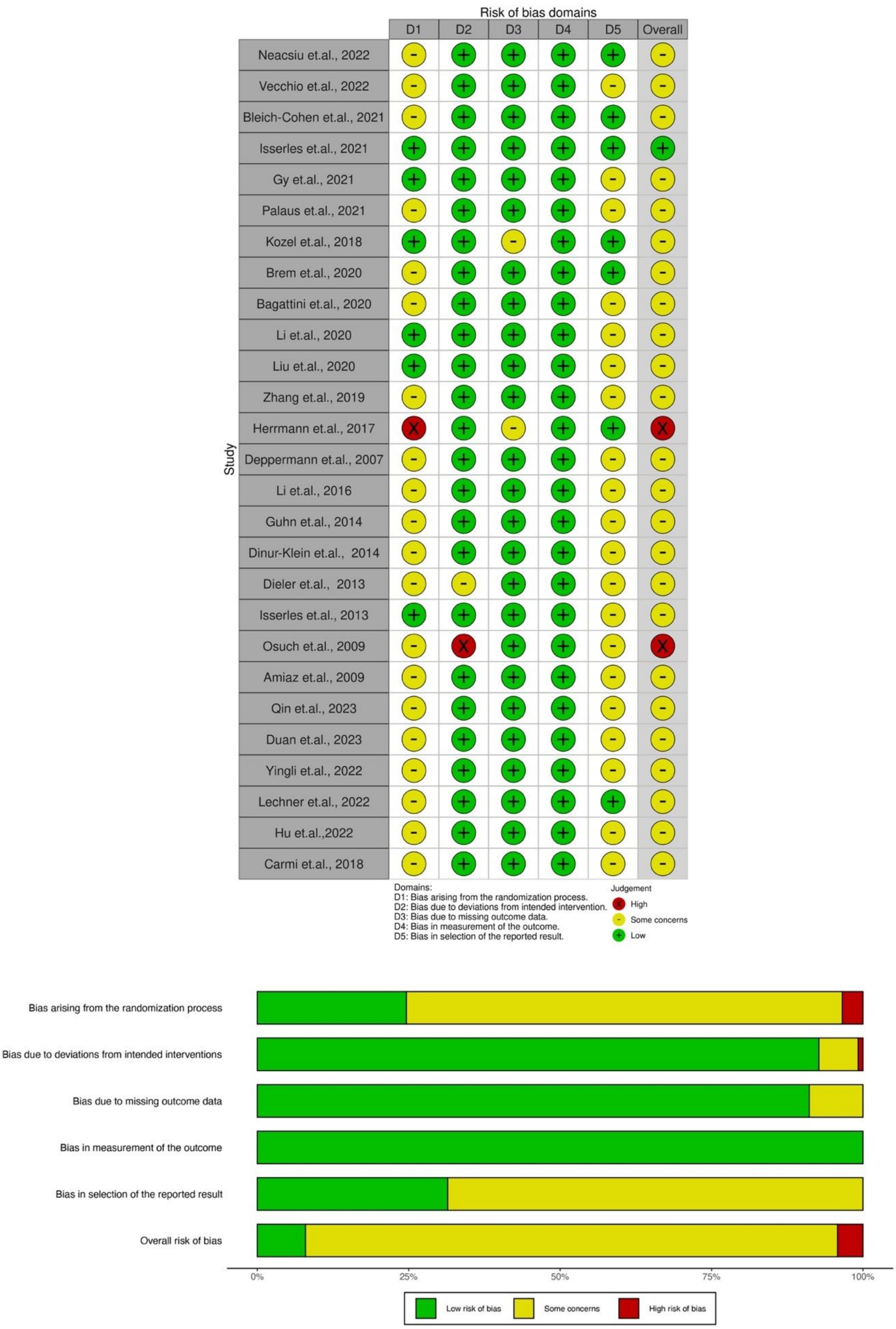

Figure S12. Risk of bias assessment. Upper figure shows each risk of bias domain for each included study and the lower figure shows percentages of each risk of bias domain across all included studies.
